# Drug Overdose Deaths Involving Stimulants ― United States, January 2018–June 2024

**DOI:** 10.15585/mmwr.mm7432a1

**Published:** 2025-08-28

**Authors:** Lauren J. Tanz, Kimberly D. Miller, Amanda T. Dinwiddie, R. Matt Gladden, Alice Asher, Grant Baldwin, Brandon Nesbit, Julie O’Donnell

**Affiliations:** 1 Division of Overdose Prevention, National Center for Injury Prevention and Control, CDC.

**Summary**

**What is already known about this topic?**

Stimulant-involved overdose deaths have increased in the United States since 2011.

**What is added by this report?**

During January 2021–June 2024, 59.0% of overdose deaths involved stimulants, 43.1% co-involved stimulants and opioids, and 15.9% involved stimulants and no opioids. Persons who died of overdoses involving stimulants and no opioids were older and more frequently had a history of cardiovascular disease than those who died of overdoses involving stimulants and opioids. Increases in stimulant-involved deaths from 2018 to 2023 were largest among non-Hispanic American Indian or Alaska Native and non-Hispanic Black or African American persons and driven by deaths co-involving stimulants and opioids.

**What are the implications for public health practice?**

Expanded access to evidence-based treatments for stimulant use disorder, evaluation of medication-based treatments, and engagement of persons using stimulants who might be missed by opioid-focused efforts might reduce deaths.

## Abstract

Drug overdose deaths involving stimulants have increased in the United States since 2011. This report describes characteristics of stimulant-involved overdose deaths during January 2021–June 2024 using CDC’s State Unintentional Drug Overdose Reporting System data and trends by drug and race and ethnicity during 2018–2023 using CDC’s National Vital Statistics System data. Overall, 59.0% of overdose deaths involved stimulants, 43.1% co-involved stimulants and opioids, and 15.9% involved stimulants and no opioids during January 2021–June 2024. Persons who died of overdoses involving stimulants and no opioids were older (aged ≥45 years; 66.5% versus 44.2%) and more frequently had a history of cardiovascular disease (38.7% versus 21.2%) than those who died of overdoses involving stimulants and opioids. Stimulant-involved overdose death rates increased from 2018 to 2023 (cocaine: 4.5 per 100,000 population to 8.6; psychostimulants with abuse potential, primarily methamphetamine: 3.9 to 10.4). Increases were largest for psychostimulants among non-Hispanic American Indian or Alaska Native persons (11.0 in 2018 to 32.9 in 2023) and cocaine among non-Hispanic Black or African American persons (9.1 to 24.3), driven by deaths co-involving stimulants and opioids. Increases in stimulant-involved deaths suggest the need for expanded access to evidence-based stimulant use disorder treatments, evaluation of medication-based treatments for stimulant use disorder and treatments for co-occurring substance use disorders, and engagement of persons who use stimulants and who might be missed by opioid-focused prevention efforts.

## Introduction

Drug overdose deaths involving stimulants, primarily cocaine and psychostimulants with abuse potential (mostly methamphetamine), have increased substantially in the United States since 2011 (*1*). The number of overdose deaths involving cocaine increased from 4,681 in 2011 to 29,449 in 2023; those involving psychostimulants with abuse potential increased from 2,266 to 34,855 (*1*). Provisional data show declines in 2024, but deaths remained well above 2011 levels.* Increases are primarily attributed to deaths co-involving opioids, although stimulant-involved deaths without opioid co-involvement have also increased (*2*). This report analyzes characteristics of stimulant-involved overdose deaths during January 2021–June 2024 using CDC’s State Unintentional Drug Overdose Reporting System (SUDORS) data and trends by drug and race and ethnicity during 2018–2023 using CDC’s National Vital Statistics System (NVSS) data.

## Methods

### Data Sources

SUDORS^†^ funds 49 states and the District of Columbia (DC) to report data on unintentional and undetermined intent drug overdose deaths from death certificates, postmortem toxicology reports, and medical examiner or coroner reports under CDC’s Overdose Data to Action in States program.^§^ SUDORS defines stimulants as cocaine, methamphetamine, other amphetamines, synthetic cathinones,^¶^ prescription stimulants,** and any other central nervous system stimulants.

NVSS^††^ collects death certificate data for all deaths in the United States and can be used to assess national trends in overdose death rates. In NVSS, overdose deaths were identified through *International Classification of Diseases, Tenth Revision* (ICD-10) underlying cause-of-death codes X40–44 (unintentional) and Y10–14 (undetermined intent) and multiple cause-of-death codes for cocaine (T40.5); psychostimulants with abuse potential (T43.6 [psychostimulants]), which primarily includes methamphetamine and other amphetamines; and opioids (T40.0–T40.4 and T40.6). In NVSS, the categories of cocaine and psychostimulants with abuse potential combined are referred to as stimulants.

### Statistical Analysis

For SUDORS data, percentages of overdose deaths during January 2021–June 2024 involving^§§^ any stimulant, and methamphetamine and cocaine as specific stimulants, were calculated and stratified by opioid co-involvement. Within strata, percentages by demographic characteristics and circumstances were calculated.

For NVSS data, annual age-adjusted rates (rates)^¶¶^ of stimulant-involved overdose deaths during 2018–2023 were calculated overall and by race and ethnicity. Analyses were performed using SAS software (version 9.4; SAS Institute). This activity was reviewed by CDC, deemed not research, and was conducted consistent with applicable federal law and CDC policy.***

## Results

### SUDORS: Stimulant-Involved Overdose Deaths During January 2021–June 2024 

**FIGURE 1:**
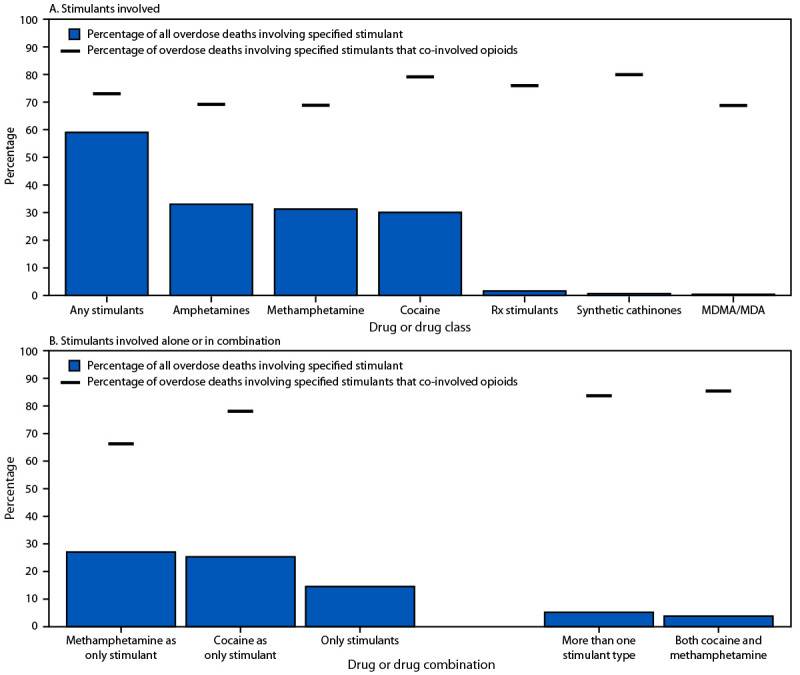
Percentage of overdose deaths (N = 309,274), by type of stimulant*^,^^†^^,^^§^^,^^¶^^,^**^,^^††^ involved^§§^ (A) and by combinations of stimulants involved (B) — State Unintentional Drug Overdose Reporting System, United States,^¶¶^ January 2021–June 2024 **Abbreviations:** MDA = 3,4-methylenedioxyamphetamine; MDMA = 3,4-methylenedioxymethamphetamine; Rx = prescription; SUDORS = State Unintentional Drug Overdose Reporting System. * Amphetamines is an overarching category that includes all amphetamines, including methamphetamine, methylenedioxymethamphetamine, and methylenedioxyamphetamine as well as all enantiomers (e.g., dextroamphetamine) and metabolites of these substances. ^†^ Prescription stimulants include armodafinil, benzphetamine, bromantane, clenbuterol, clobenzorex, dexmethylphenidate, diethylpropion, lisdexamfetamine, mephentermine, methylphenidate, modafinil, phendimetrazine, phentermine, propylhexedrine, and ritalinic acid. This category also includes amphetamine (including 4-hydroxyamphetamine and dextroamphetamine) if methamphetamine is not detected, levoamphetamine if dextroamphetamine is not detected, and levomethamphetamine if dextromethamphetamine is not detected. ^§^ Synthetic cathinones are laboratory-made stimulants chemically related to substances found in the khat plant. ^¶^ The methamphetamine as only stimulant and cocaine as only stimulant categories could have had other nonstimulant drugs (e.g., opioids) involved. ** The only stimulants category includes deaths in which only stimulants were considered to have caused or contributed to the death; other drugs could have been detected but were not considered to have contributed to the death. ^††^ The more than one stimulant type category includes deaths in which stimulants in more than one of the following categories were considered to have caused or contributed to the death: methamphetamine, cocaine, prescription stimulants, cathinones, MDMA/MDA, or other stimulants (e.g., phenethylamine). ^§§^A drug was classified as involved or co-involved in overdose deaths if the medical examiner or coroner listed it as causing death on the death certificate, or in the medical examiner or coroner report or postmortem toxicology report. ^¶¶^ Includes 49 states and the District of Columbia. For inclusion, jurisdictions were required to report ≥75% of overdose deaths in their jurisdiction in at least one 6-month period during January 2021–June 2024. All jurisdictions except four (California, Texas, Wisconsin, and Wyoming) reported deaths for the full time frame. California and Wisconsin were included for a subset of the time frame. Texas and Wyoming were only funded for SUDORS starting with deaths that occurred during January–June 2024. North Dakota was not included because it is not funded for SUDORS.

**Types of stimulants involved. **Among 309,274 overdose deaths during January 2021–June 2024 in 49 states and DC,^†††^ 59.0% involved any stimulant, 31.2% involved methamphetamine, and 30.0% involved cocaine; 3.8% involved both methamphetamine and cocaine (Figure 1). Prescription stimulants (1.6%), synthetic cathinones (0.7%), and 3,4-methylenedioxymethamphetamine (MDMA)/3,4-methylenedioxyamphetamine (MDA) (0.4%) were rarely involved. Most (73.0%) stimulant-involved overdose deaths co-involved opioids, including 79.1% of cocaine-involved and 68.8% of methamphetamine-involved deaths; 14.5% of deaths involved only stimulants.

**Characteristics of stimulant-involved overdose deaths**. Overall, 182,502 (59.0%) overdose deaths involved stimulants, 133,293 (43.1%) co-involved stimulants and opioids, and 49,209 (15.9%) involved stimulants and no opioids (Table). Across stimulant and opioid groups, approximately 70% of decedents were male, and 30% were female. A higher percentage of persons who died of overdoses involving stimulants and no opioids was aged ≥45 years (66.5%) than of those who died of overdoses co-involving stimulants and opioids (44.2%).

**TABLE:** Number and percentage of overdose deaths involving stimulants and opioids,* by select decedent characteristics and overdose circumstances ― State Unintentional Drug Overdose Reporting System, United States, January 2021–June 2024

Characteristic	No. (%)									
	Total overdose deaths	Total stimulant-involved overdose deaths^†^			Cocaine-involved overdose deaths			Methamphetamine-involved overdose deaths		
		Total	Opioids co-involved	Opioids not involved	Total	Opioids co-involved	Opioids not involved	Total	Opioids co-involved	Opioids not involved
**Overall (49 states and DC)^§,¶^**	**N = 309,274**	**n = 182,502 (59.0%)**	**n = 133,293 (43.1%)**	**n = 49,209 (15.9%)**	**n = 92,697 (30.0%)**	**n = 73,292 (23.7%)**	**n = 19,405 (6.3%)**	**n = 96,614 (31.2%)**	**n = 66,475 (21.5%)**	**n = 30,139 (9.7%)**
Age group, yrs**										
<15	**836 (0.3)**	**146 (0.1)**	70 (0.1)	76 (0.2)	**52 (0.1)**	36 (<0.1)	16 (0.1)	**91 (0.1)**	34 (0.1)	57 (0.2)
15–24	**17,042 (5.5)**	**6,597 (3.6)**	5,659 (4.2)	938 (1.9)	**3,204 (3.5)**	2,876 (3.9)	328 (1.7)	**3,449 (3.6)**	2,879 (4.3)	570 (1.9)
25–34	**64,535 (20.9)**	**34,860 (19.1)**	29,517 (22.1)	5,343 (10.9)	**16,485 (17.8)**	14,534 (19.8)	1,951 (10.1)	**19,577 (20.3)**	16,246 (24.4)	3,331 (11.1)
35–44	**81,098 (26.2)**	**49,257 (27.0)**	39,112 (29.3)	10,145 (20.6)	**22,901 (24.7)**	19,626 (26.8)	3,275 (16.9)	**28,350 (29.4)**	21,534 (32.4)	6,816 (22.6)
45–54	**65,527 (21.2)**	**41,972 (23.0)**	29,339 (22.0)	12,633 (25.7)	**21,297 (23.0)**	16,755 (22.9)	4,542 (23.4)	**22,323 (23.1)**	14,147 (21.3)	8,176 (27.1)
55–64	**59,721 (19.3)**	**38,307 (21.0)**	23,760 (17.8)	14,547 (29.6)	**22,003 (23.7)**	15,537 (21.2)	6,466 (33.3)	**17,898 (18.5)**	9,498 (14.3)	8,400 (27.9)
≥65	**20,463 (6.6)**	**11,326 (6.2)**	5,816 (4.4)	5,510 (11.2)	**6,744 (7.3)**	3,920 (5.3)	2,824 (14.6)	**4,899 (5.1)**	2,125 (3.2)	2,774 (9.2)
Sex**										
Female	**89,029 (28.8)**	**50,936 (27.9)**	37,743 (28.3)	13,193 (26.8)	**25,612 (27.6)**	20,406 (27.8)	5,206 (26.8)	**26,871 (27.8)**	18,968 (28.5)	7,903 (26.2)
Male	**220,231 (71.2)**	**131,561 (72.1)**	95,545 (71.7)	36,016 (73.2)	**67,082 (72.4)**	52,883 (72.2)	14,199 (73.2)	**69,739 (72.2)**	47,503 (71.5)	2,236 (73.8)
Race and ethnicity**										
American Indian or Alaska Native, NH	**4,557 (1.5)**	**2,819 (1.6)**	1,820 (1.4)	1,071 (2.2)	**623 (0.7)**	480 (0.7)	143 (0.7)	**2,372 (2.5)**	1,420 (2.2)	952 (3.2)
Asian, NH	**2,429 (0.8)**	**1,483 (0.8)**	876 (0.7)	607 (1.2)	**706 (0.8)**	502 (0.7)	204 (1.1)	**784 (0.8)**	408 (0.6)	376 (1.3)
Black or African American, NH	**64,655 (21.1)**	**41,764 (23.2)**	30,362 (23.0)	11,402 (23.5)	**33,990 (37.1)**	25,362 (35.0)	8,628 (45.0)	**9,966 (10.4)**	6,946 (10.6)	3,020 (10.1)
Native Hawaiian or Pacific Islander, NH	**382 (0.1)**	**280 (0.2)**	115 (0.1)	165 (0.3)	**44 (<0.1)**	33 (<0.1)	11 (0.1)	**244 (0.3)**	87 (0.1)	157 (0.5)
White, NH	**194,730 (63.6)**	**110,822 (61.4)**	81,629 (61.9)	29,193 (60.1)	**43,575 (47.6)**	35,873 (49.5)	7,702 (40.2)	**70,896 (74.2)**	49,414 (75.1)	21,482 (72.2)
Multiple races, NH	**3,565 (1.2)**	**2,212 (1.2)**	1,431 (1.1)	781 (1.6)	**744 (0.8)**	601 (0.8)	143 (0.7)	**1,537 (1.6)**	895 (1.4)	642 (2.2)
Hispanic or Latino	**35,813 (11.7)**	**20,953 (11.6)**	15,589 (11.8)	5,364 (11.0)	**11,902 (13.0)**	9,572 (13.2)	2,330 (12.2)	**9,762 (10.2)**	6,633 (10.1)	3,129 (10.5)
**Among deaths with circumstance data (46 states and DC)^¶,^** ^††,^ **^§§,¶¶^**	**N = 226,698**	**n = 134,109 (59.2%)**	**n = 99,534 (43.9%)**	**n = 34,575 (15.3%)**	**n = 71,622 (31.6%)**	**n = 57,567 (25.4%)**	**n = 14,055 (6.2%)**	**n = 67,831 (29.9%)**	**n = 46,985 (20.7%)**	**n = 20,846 (9.2%)**
**Medical history**										
Cardiovascular disease***	**55,305 (24.4)**	**34,467 (25.7)**	21,084 (21.2)	13,383 (38.7)	**18,159 (25.4)**	12,805 (22.2)	5,354 (38.1)	**17,488 (25.8)**	9,288 (19.8)	8,200 (39.3)
Obesity	**28,099 (12.4)**	**15,485 (11.5)**	10,842 (10.9)	4,643 (13.4)	**8,063 (11.3)**	6,288 (10.9)	1,775 (12.6)	**7,835 (11.6)**	4,942 (10.5)	2,893 (13.9)
**History of drug use**										
Any drugs	**176,546 (77.9)**	**106,071 (79.1)**	80,414 (80.8)	25,657 (74.2)	**55,692 (77.8)**	45,304 (78.7)	10,388 (73.9)	**54,960 (81.0)**	39,304 (83.7)	15,656 (75.1)
Stimulants^†††^	**59,367 (26.2)**	**46,840 (34.9)**	31,362 (31.5)	15,478 (44.8)	**24,394 (34.1)**	18,209 (31.6)	6,185 (44.0)	**24,916 (36.7)**	15,238 (32.4)	9,678 (46.4)
Cocaine	**34,972 (15.4)**	**26,638 (19.9)**	19,630 (19.7)	7,008 (20.3)	**23,174 (32.4)**	17,179 (29.8)	5,995 (42.7)	**5,472 (8.1)**	4,105 (8.7)	1,367 (6.6)
Methamphetamine	**29,257 (12.9)**	**24,033 (17.9)**	14,515 (14.6)	9,518 (27.5)	**3,083 (4.3)**	2,481 (4.3)	602 (4.3)	**22,331 (32.9)**	13,193 (28.1)	9,138 (43.8)
Opioids^§§§^	**77,025 (34.0)**	**39,607 (29.5)**	35,601 (35.8)	4,006 (11.6)	**19,434 (27.1)**	17,965 (31.2)	1,469 (10.5)	**21,876 (32.3)**	19,333 (41.1)	2,543 (12.2)
**Mental health and substance use disorders**										
Evidence of a mental health diagnosis	**63,046 (27.8)**	**34,629 (25.8)**	26,299 (26.4)	8,330 (24.1)	**17,645 (24.6)**	14,443 (25.1)	3,202 (22.8)	**17,963 (26.5)**	12,882 (27.4)	5,081 (24.4)
Ever treated for mental health or substance use disorders^¶¶¶^	**54,978 (24.3)**	**28,452 (21.2)**	23,300 (23.4)	5,152 (14.9)	**15,236 (21.3)**	13,084 (22.7)	2,152 (15.3)	**14,208 (20.9)**	11,253 (24.0)	2,955 (14.2)
Current treatment for mental health or substance use disorders^¶¶¶^	**33,301 (14.7)**	**16,111 (12.0)**	13,152 (13.2)	2,959 (8.6)	**8,963 (12.5)**	7,743 (13.5)	1,220 (8.7)	**7,404 (10.9)**	5,743 (12.2)	1,661 (8.0)
Ever treated for substance use disorders^¶¶¶^	**34,807 (15.4)**	**18,270 (13.6)**	15,824 (15.9)	2,446 (7.1)	**9,723 (13.6)**	8,686 (15.1)	1,037 (7.4)	**9,487 (14.0)**	8,078 (17.2)	1,409 (6.8)
Current treatment for substance use disorders^¶¶¶^	**14,856 (6.6)**	**7,084 (5.3)**	6,243 (6.3)	841 (2.4)	**4,064 (5.7)**	3,693 (6.4)	371 (2.6)	**3,303 (4.9)**	2,834 (6.0)	469 (2.3)
Experiencing homelessness or housing instability****	**24,113 (11.0)**	**17,639 (13.5)**	13,517 (14.0)	4,122 (12.2)	**6,804 (9.8)**	5,824 (10.4)	980 (7.2)	**11,827 (17.9)**	8,606 (18.9)	3,221 (15.7)
**Overdose circumstances**										
Overdose occurred where they lived	**132,561 (60.3)**	**72,699 (55.7)**	52,978 (54.8)	19,721 (58.4)	**40,307 (57.4)**	31,625 (56.0)	8,682 (63.1)	**34,532 (52.8)**	23,325 (51.8)	11,207 (55.1)
Naloxone administered^††††^	**51,539 (22.7)**	**29,559 (22.0)**	24,156 (24.3)	5,403 (15.6)	**14,937 (20.9)**	12,719 (22.1)	2,218 (15.8)	**15,964 (23.5)**	12,719 (27.1)	3,245 (15.6)
Potential bystanders present^§§§§^	**98,772 (43.6)**	**57,515 (42.9)**	43,289 (43.5)	14,226 (41.2)	**29,957 (41.8)**	24,425 (42.4)	5,532 (39.4)	**29,708 (43.8)**	20,927 (44.6)	8,781 (42.1)
No pulse at first responder arrival	**142,484 (63.8)**	**85,339 (64.6)**	64,433 (65.7)	20,906 (61.3)	**44,812 (63.4)**	36,529 (64.3)	8,283 (59.6)	**44,096 (66.1)**	31,231 (67.7)	12,865 (62.6)
Seen in emergency department^¶¶¶¶^	**48,254 (21.7)**	**29,205 (22.2)**	17,939 (18.4)	11,266 (33.1)	**15,591 (22.1)**	10,573 (18.7)	5,018 (36.4)	**14,233 (21.4)**	7,989 (17.4)	6,244 (30.3)

**Abbreviations:** DC = District of Columbia; NH = non-Hispanic; SUDORS = State Unintentional Drug Overdose Reporting System.

* A drug was classified as involved or co-involved in overdose deaths if the medical examiner or coroner listed it as cause of death on the death certificate, or in the medical examiner or coroner report or postmortem toxicology report. Because deaths might involve both cocaine and methamphetamine, some deaths are included in both categories; categories across stimulant types are not mutually exclusive. Within stimulant types, categories with and without opioid co-involvement are mutually exclusive (e.g., among cocaine-involved overdose deaths “opioids co-involved” is mutually exclusive from “opioids not involved”).

^†^ Includes deaths involving cocaine, methamphetamine, and any other stimulants (e.g., synthetic cathinones (laboratory-made stimulants chemically related to substances found in the khat plant), prescription dextroamphetamine/amphetamine, 3,4-methylenedioxymethamphetamine) involved in overdose deaths.

^§^ For inclusion, jurisdictions were required to report ≥75% of overdose deaths in their jurisdiction in at least one 6-month period during January 2021–June 2024. All jurisdictions except four (California, Texas, Wisconsin, and Wyoming) reported deaths for the full time frame. California and Wisconsin were included for a subset of the time frame. Texas and Wyoming were only funded for SUDORS starting with deaths that occurred during January–June 2024. North Dakota was not included because it is not funded for SUDORS.

^¶^ Percentages in rows titled “overall” and “among deaths with circumstance data” are row percentages. All other percentages are column percentages.

** Missing values were excluded from calculations of percentages. Thus, the counts of each category might not sum to the total due to missing data. Percentages might not sum to 100 because of rounding. Overall, <0.1% of deaths were missing age, <0.1% were missing sex, and 1.0% were missing race and ethnicity.

^††^ Includes Alabama, Alaska, Arizona, Arkansas, Colorado, Connecticut, Delaware, DC, Florida, Georgia, Hawaii, Illinois, Indiana, Iowa, Kansas, Kentucky, Louisiana, Maine, Massachusetts, Maryland, Michigan, Minnesota, Mississippi, Missouri, Montana, Nebraska, Nevada, New Hampshire, New Jersey, New Mexico, New York, North Carolina, Ohio, Oklahoma, Oregon, Pennsylvania, Rhode Island, South Carolina, South Dakota, Tennessee, Utah, Vermont, Virginia, Washington, West Virginia, Wisconsin, and Wyoming.

^§§^ For inclusion, jurisdictions were required to report ≥75% of overdose deaths in their jurisdiction and have circumstance data for ≥75% of overdose deaths in at least one 6-month period during January 2021–June 2024. Thirty-two jurisdictions met the requirements for the full time frame; 15 met the requirements for at least one 6-month period. Among the 47 included jurisdictions, 93.1% of deaths had data on circumstances and were included in analyses. Demographic characteristics of deaths included in this sample are similar to demographic characteristics in the sample including all deaths.

^¶¶^ Missing values were excluded from calculations of percentages. Percentage missing for each variable is as follows: ever treated for substance use disorders, <0.1%; current treatment for substance use disorders, <0.1%; experiencing homelessness or housing instability, 2.9%; overdose occurred where they lived, 3.0%; naloxone administered, <0.1%; potential bystanders present, <0.1%; no pulse at first responder arrival, 1.5%; seen in emergency department, 2.0%. All other variables had no missing data.

*** In SUDORS, history of cardiovascular disease excludes hypertension.

^†††^ Includes history of cocaine or methamphetamine use.

^§§§^ Includes history of any opioid use, such as prescription opioid misuse or use of heroin or illegally manufactured fentanyls.

^¶¶¶^ Treatment for substance use disorders includes medications for opioid use disorder, living in an inpatient rehabilitation facility, participating in mental health or substance use disorder outpatient treatment, or attending Narcotics Anonymous or Alcoholics Anonymous (if specified to be for drug use).

**** Persons experiencing homelessness were those who lived in places not designed for or ordinarily used as regular sleeping accommodations or in a supervised shelter or drop-in center designated to provide temporary living arrangements, congregate shelters, or temporary accommodations provided by a homeless shelter. Persons experiencing housing instability were not experiencing homelessness but lacked the resources or support networks to obtain or retain permanent housing, including interrelated challenges such as trouble paying rent, overcrowding, moving frequently, or staying with relatives.

^††††^ Naloxone is a life-saving medication that can reverse an overdose from opioids, including heroin, fentanyl, and prescription opioids. Because SUDORS only includes people who died of a drug overdose, evidence of naloxone administration reflects failed administration (i.e., it did not reverse the overdose). This could be because naloxone was not administered soon enough or in sufficient dosages to reverse the overdose, it was not effective (i.e., overdose did not involve opioids), or its effectiveness was affected by polydrug use.

^§§§§^ A potential bystander is defined as a person aged ≥11 years who was physically nearby during or shortly preceding an overdose and potentially had an opportunity to intervene or respond. This includes persons in the same structure (e.g., same room or same building, but different room) as the decedent during that time. This does not include persons in different self-contained parts of buildings (e.g., persons in a different apartment in the same apartment building would not be a potential bystander).

^¶¶¶¶^ Denotes whether the person was seen in an emergency department for the fatal overdose regardless of whether they were dead or alive on arrival and regardless of whether or not they received treatment.

Among 46 states and DC^§§§^ with sufficient circumstance data,^¶¶¶^ higher percentages of persons who died of overdoses involving stimulants and no opioids had a known history of cardiovascular disease (CVD) compared with those who died of overdoses co-involving stimulants and opioids (38.7% versus 21.2%) and were seen in the emergency department (ED) for the fatal overdose (33.1% versus 18.4%). Lower percentages of persons who died of overdoses involving stimulants and no opioids, compared with those co-involving stimulants and opioids, had evidence of opioid use history (11.6% versus 35.8%) and of ever receiving treatment for mental health or substance use disorders (SUDs) (14.9% versus 23.4%). For deaths specifically involving cocaine or methamphetamine, with or without opioid co-involvement, patterns were comparable to those for deaths involving any stimulant.

### NVSS: Trends in Stimulant-Involved Overdose Death Rates from 2018 to 2023

**FIGURE 2:**
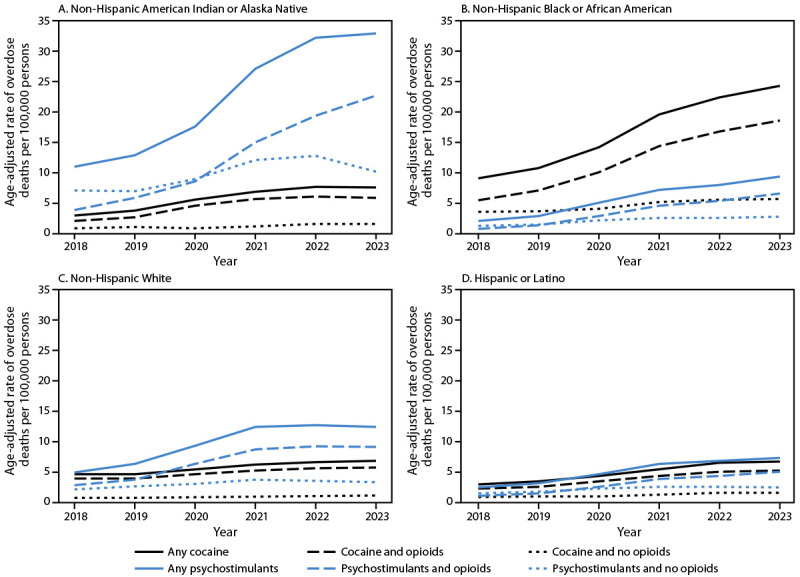
Age-adjusted rates* of overdose deaths^†^ involving stimulants and co-involving opioids,^§^^,^^¶^ by race and ethnicity** and year of death — National Vital Statistics System, United States, 2018–2023 **Abbreviation**: ICD-10 = *International Classification of Diseases, Tenth Revision.* * Age-adjusted death rates were calculated by weighting age-specific rates per 100,000 vintage year population to the 2000 U.S. Census Bureau standard population. ^†^ Overdose deaths were identified through ICD-10 underlying cause-of-death codes X40–44 (unintentional) and Y10–14 (undetermined intent). ^§^ Drug categories were defined using multiple cause-of-death ICD-10 codes: cocaine (T40.5), psychostimulants with abuse potential (T43.6), and opioids (T40.0–T40.4 and T40.6). ^¶^ Because deaths might involve both cocaine and psychostimulants, some deaths are included in both categories. ** Overall data and data for non-Hispanic Asian and non-Hispanic Native Hawaiian or Pacific Islander persons are shown in the Supplementary Figure.

Overall, 520,525 unintentional and undetermined intent overdose deaths occurred during 2018–2023 in the U.S. Overdose death rates per 100,000 population involving cocaine and psychostimulants with abuse potential increased from 2018 to 2023 (cocaine: 4.5 to 8.6; psychostimulants: 3.9 to 10.4) (Supplementary Figure). Among non-Hispanic American Indian or Alaska Native (AI/AN) persons, the rate of overdose deaths involving psychostimulants increased from 11.0 in 2018 to 32.9 in 2023 and was higher than the cocaine-involved death rate throughout the time frame (Figure 2). Among non-Hispanic Black or African American (Black) persons, the cocaine-involved death rate increased from 9.1 in 2018 to 24.3 in 2023 and exceeded the psychostimulant-involved death rate throughout. In addition, the psychostimulant-involved death rate increased from 7.4 in 2018 to 16.3 in 2023 among non-Hispanic Native Hawaiian or Pacific Islander persons. Although stimulant-involved death rates were lower across the time frame for non-Hispanic White, non-Hispanic Asian, and Hispanic or Latino persons, the rates also increased from 2018 to 2023. Across groups, the largest increases were in deaths co-involving stimulants and opioids.

## Discussion

Although most overdose deaths involve opioids (*1*), 182,502 persons died of overdoses involving stimulants (with or without other drugs co-involved) in the United States during January 2021–June 2024, accounting for 59% of all overdose deaths. From 2018 to 2023, the largest increases were in deaths co-involving stimulants and opioids, although types of stimulants involved and corresponding increases, varied by race and ethnicity. Persons who died of overdoses involving stimulants and no opioids differed (e.g., were older and more often had a history of CVD) from those who died of overdoses co-involving stimulants and opioids.

Stimulant overdoses often present as cardiovascular complications (e.g., stroke), disrupt thermoregulation, and cause mental health symptoms (*3*,*4*); long-term stimulant use is associated with CVD, increasing stimulant overdose risk (*4*). Correspondingly, 38.7% of persons who died of overdoses involving stimulants and no opioids, and 21.2% of those who died of overdoses involving both stimulants and opioids, had a known history of CVD (versus approximately 10% among all U.S. adults) (*5*). Assessing stimulant use during health care visits might identify persons at increased risk for stimulant-induced cardiovascular events (*4*).

Teaching persons who use drugs and their family and friends, as well as medical professionals, how to identify symptoms of stimulant overdoses (e.g., CVD events or psychosis) might improve response timeliness and save lives. Unlike opioids, no stimulant overdose reversal agents or approved medication-based treatments for stimulant use disorder exist (*3*). Contingency management, a behavioral intervention that positively reinforces recovery-related behaviors (e.g., abstinence), is the most effective treatment for stimulant use disorder but remains underused**** (*4*). Low-barrier care models and improved linkage to care (e.g., during ED visits for nonfatal overdoses involving stimulants) might increase treatment uptake and retention.^††††^

Seventy three percent of stimulant-involved overdose deaths co-involved opioids, representing almost 45% of all overdose deaths. Drug-checking programs rarely detected opioids in recent stimulant products, suggesting that persons who died of overdoses involving both stimulants and opioids intentionally co-used separate stimulant and opioid products (*6*,*7*). Reasons persons have reported for co-using opioids and stimulants include reducing opioid withdrawal symptoms and balancing the sedating effects of opioids; despite perceived benefits, co-use can increase overdose risk (*3*). In the event of an overdose, opioid reversal agents do not reverse stimulant effects; therefore, additional treatment of stimulant effects (e.g., treatment for agitation or seizures and implementation of cooling strategies) might be needed when opioids and stimulants are used together (*3*). In addition, stimulant use complicates opioid use disorder (OUD) treatment, and guidance is limited for treating co-occurring stimulant use disorder and OUD (*4*). Coordinated, evidence-based treatments for co-occurring use disorders are urgently needed. Provisional data indicating declines in overdose deaths in 2024 primarily reflect decreases in deaths involving synthetic opioids such as illegally manufactured fentanyls (IMFs) (*8*); challenges treating co-occurring stimulant use disorder and OUD might attenuate declines.

Approximately 50,000 (15%) overdose deaths during 3.5 years involved stimulants and no opioids. Nearly 40% had a CVD history, possibly representing long-term stimulant use (*4*). Persons who use stimulants but not opioids have limited tailored interventions and treatments available to prevent death and support recovery and might be missed by existing opioid-focused prevention efforts. Including stimulant-specific guidance in risk reduction strategies and improving access to and retention in treatment for stimulant use disorder might reduce overdose deaths. In addition, persons who died of overdoses involving stimulants and no opioids were older and less often had an opioid use history than those who died of overdoses co-involving stimulants and opioids; overdose prevention and response efforts might therefore require different outreach strategies to reach this population.

From 2018 to 2023, AI/AN persons experienced the largest increases in psychostimulant-involved overdose death rates, and Black persons experienced the largest increases in cocaine-involved death rates. During this time, the prevalence of methamphetamine use among AI/AN persons, cocaine use among Black persons, and opioid use and misuse in both groups remained relatively stable.^§§§§^ This suggests increased drug potency or differential access to health care and treatments for SUDs among these groups (*9*). Compared with eastern U.S. drug markets, IMFs proliferated later in southern markets where Black persons disproportionately live, and in western markets where AI/AN persons disproportionately live (*10*); recent increases in deaths co-involving stimulants and opioids among these groups might reflect these market changes. Prevention messaging addressing specific stimulants commonly involved in different populations and improved engagement and retention in care might decrease deaths.

### Limitations

The findings in this report are subject to at least five limitations. First, given cardiovascular outcomes, some stimulant-involved overdose deaths might be certified such that they do not receive overdose-specific ICD-10 codes (e.g., cause of death listed as acute cerebrovascular event in setting of methamphetamine toxicity) and thus be missed. Second, postmortem toxicology testing varies within and across jurisdictions, and testing for emerging stimulants is not routine. Together, these factors likely contribute to an underestimation of stimulant-involved overdose deaths. Third, circumstance information depends on availability in investigative reports; percentages are likely underestimated. Fourth, race and ethnicity might be misclassified, potentially affecting race- and ethnicity-specific death rates. Finally, although analyses included nearly all states, some large states were excluded from SUDORS circumstance analyses, reducing generalizability.

### Implications for Public Health Practice

Nearly 60% of overdose deaths during January 2021–June 2024 involved stimulants, highlighting the need for expanded access to evidence-based behavioral treatments (e.g., contingency management) for stimulant use disorder, additional evaluation of medication-based treatments for stimulant use disorder and treatments for co-occurring use disorders, and increased retention in care. In addition, because the signs and symptoms of stimulant overdoses differ from those of opioid overdoses, teaching persons who use drugs and their family and friends how to recognize and respond to stimulant overdoses might save lives. Persons who died from overdoses involving stimulants and no opioids were different (e.g., were older and more often had a history of CVD) from those who died from overdoses co-involving stimulants and opioids and might be missed by opioid-focused prevention efforts. Expanding prevention efforts focused on stimulant overdoses might reduce deaths.

## Footnotes

* Products - Vital Statistics Rapid Release - Provisional Drug Overdose Death Counts (Accessed May 14, 2025).

† About the State Unintentional Drug Overdose Reporting System (SUDORS) | Overdose Prevention | CDC (Accessed April 14, 2025). For SUDORS, overdose deaths were identified by funded jurisdictions both through ICD-10 underlying cause-of-death codes X40–44 (unintentional) and Y10–14 (undetermined intent) and through text searches of literal cause of death fields on death certificates that indicated acute drug toxicity.

§ Overdose Data to Action in States | Overdose Prevention | CDC (Accessed April 14, 2025).

¶ Synthetic cathinones are laboratory-made stimulants chemically related to substances found in the khat plant. Synthetic Cathinones (“Bath Salts”) | National Institute on Drug Abuse (NIDA)

** Prescription stimulants include armodafinil, benzphetamine, bromantane, clenbuterol, clobenzorex, dexmethylphenidate, diethylpropion, lisdexamfetamine, mephentermine, methylphenidate, modafinil, phendimetrazine, phentermine, propylhexedrine, and ritalinic acid. This category also includes amphetamine (including 4-hydroxyamphetamine and dextroamphetamine) if methamphetamine is not detected, levoamphetamine if dextroamphetamine is not detected, and levomethamphetamine if dextromethamphetamine is not detected.

†† NVSS - Mortality Statistics (Accessed April 14, 2025).

§§ A drug was classified as involved or co-involved in overdose deaths if the medical examiner or coroner listed it as cause of death on the death certificate or in the medical examiner or coroner report or postmortem toxicology report.

¶¶ Age-adjusted death rates were calculated by weighting age-specific rates per 100,000 vintage year population to the 2000 U.S. Census Bureau standard population.

*** 45 C.F.R. part 46.102(l)(2), 21 C.F.R. part 56; 42 U.S.C. Sect. 241(d); 5 U.S.C. Sect. 552a; 44 U.S.C. Sect. 3501 et seq.

††† For inclusion, jurisdictions were required to report ≥75% of overdose deaths in their jurisdiction in at least one 6-month period during January 2021–June 2024. All jurisdictions except four (California, Texas, Wisconsin, and Wyoming) reported deaths for the full time frame. California and Wisconsin were included for a subset of the time frame. Texas and Wyoming were only funded for SUDORS starting with deaths that occurred during January–June 2024. North Dakota was not included because it is not funded for SUDORS.

§§§ Alabama, Alaska, Arizona, Arkansas, Colorado, Connecticut, Delaware, DC, Florida, Georgia, Hawaii, Illinois, Indiana, Iowa, Kansas, Kentucky, Louisiana, Maine, Massachusetts, Maryland, Michigan, Minnesota, Mississippi, Missouri, Montana, Nebraska, Nevada, New Hampshire, New Jersey, New Mexico, New York, North Carolina, Ohio, Oklahoma, Oregon, Pennsylvania, Rhode Island, South Carolina, South Dakota, Tennessee, Utah, Vermont, Virginia, Washington, West Virginia, Wisconsin, and Wyoming.

¶¶¶ For inclusion, jurisdictions were required to report ≥75% of overdose deaths in their jurisdiction and have circumstance data for ≥75% of overdose deaths in at least one 6-month period during January 2021–June 2024. Thirty-two jurisdictions met the requirements for the full time frame; 15 met the requirements for at least one 6-month period. Among the 47 included jurisdictions, 93.1% of deaths had data on circumstances and were included in analyses. Demographic characteristics of deaths included in this sample are similar to demographic characteristics in the sample including all deaths.

**** Contingency Management for the Treatment of Substance Use Disorders: Enhancing Access, Quality, and Program Integrity for an Evidence-Based Intervention (Accessed April 14, 2025).

†††† Advisory: Low Barrier Models of Care for Substance Use Disorders; Public Safety-Led Post-Overdose Outreach (Accessed April 23, 2025).

§§§§ 2018 NSDUH Detailed Tables | CBHSQ Data; 2019 NSDUH Detailed Tables | CBHSQ Data; 2020 NSDUH Detailed Tables | CBHSQ Data; 2021 NSDUH Detailed Tables | CBHSQ Data; 2022 NSDUH Detailed Tables | CBHSQ Data; 2023 NSDUH Detailed Tables | CBHSQ Data (Accessed April 14, 2025).
